# Bayesian pattern-mixture models for dropout and intermittently missing data in longitudinal data analysis

**DOI:** 10.3758/s13428-023-02128-y

**Published:** 2023-05-23

**Authors:** Shelley A. Blozis

**Affiliations:** Department of Psychology, University of California, Davis, Davis, CA USA

**Keywords:** Nonignorable missingness, Nonlinear mixed-effects models, Three-level hierarchical models

## Abstract

Valid inference can be drawn from a random-effects model for repeated measures that are incomplete if whether the data are missing or not, known as missingness, is independent of the missing data. Data that are missing completely at random or missing at random are two data types for which missingness is ignorable. Given ignorable missingness, statistical inference can proceed without addressing the source of the missing data in the model. If the missingness is not ignorable, however, recommendations are to fit multiple models that represent different plausible explanations of the missing data. A popular choice in methods for evaluating nonignorable missingness is a random-effects pattern-mixture model that extends a random-effects model to include one or more between-subjects variables that represent fixed patterns of missing data. Generally straightforward to implement, a fixed pattern-mixture model is one among several options for assessing nonignorable missingness, and when it is used as the sole model to address nonignorable missingness, understanding the impact of missingness is greatly limited. This paper considers alternatives to a fixed pattern-mixture model for nonignorable missingness that are generally straightforward to fit and encourage researchers to give greater attention to the possible impact of nonignorable missingness in longitudinal data analysis. Patterns of both monotonic and non-monotonic (intermittently) missing data are addressed. Empirical longitudinal psychiatric data are used to illustrate the models. A small Monte Carlo data simulation study is presented to help illustrate the utility of such methods.

**Supplementary Information:**

The online version contains supplementary material available at 10.3758/s13428-023-02128-y.

Psychological and behavioural data are gathered at multiple points in time to study how variables change or develop. Despite efforts to obtain complete data in a longitudinal study, missing data can sometimes be unavoidable. Further, measures can be taken at different times for different subjects, making the data analysis complex. As random-effects models naturally allow for responses to be observed at different points in time between subjects, the models naturally handle missing response data. If some values are missing, valid inference depends on whether the missingness (i.e., whether or not the data are missing) is independent of the missing response (Laird, 1988). If independent, the missingness is ignorable. Missingness is not ignorable, however, if the missingness is related to the missing data, even if after conditioning on model covariates (Little & Rubin, 2002).

A potentially serious problem with nonignorable missingness is that model inference can be biased, making it essential to address the missingness (Little & Rubin, 2002). For example, if the data for participants who drop from a study tend to differ from those who completed the study, then accounting for subject attrition is informative when modelling the longitudinal data. A problem in evaluating the mechanism giving rise to missing data, however, is that any model applied to empirical data is sensitive to unverifiable assumptions. Indeed, when drawing inference from a random-effects model, certainty about whether the missingness is ignorable or not is problematic because the analyst has access to only the observed data, but ignorable missingness involves the missing data. Further, the fit of a given model to data that are not complete is based on how well the model fits the observed data and not the unobserved data, creating a challenge in efforts to assess possible nonignorable missingness. For these reasons, recommendations include fitting multiple models that represent plausible explanations of the missing data in a given application (Molenberghs & Kenward, 2007). Importantly, the fit of any model is not testable given that only observed data are available for analysis. Inference may proceed by making comparisons between models that differ in their assumptions about the missing data process, while assessing the sensitivity of inference about the longitudinal process under different but plausible mechanisms for the missing data.

## Missingness in longitudinal data

Several approaches are available for addressing missingness in longitudinal data, and before describing some of the major frameworks for this, it is useful to introduce notation for the data model of a longitudinal outcome and a separate model for non-response. Consider longitudinal data for a normal outcome variable ***Y***_*i*_ = (*Y*_*i*1_, …, *Y*_*in*_)^′^, where measures for all *i* = 1, …, *N* individuals are planned for *n* occasions. Interest in ***Y***_*i*_ often concerns how the response depends on time and possibly covariates that may vary by occasion or the individual, and thus, a data model is generated based on these considerations. Letting ***X***_*i*_ be an *n* × *p* matrix that contains study design information (e.g., measures of time when ***Y***_*i*_ was observed and covariates), the multivariate density of ***Y***_*i*_ is conditional on ***X***_*i*_ and a set of unknown parameters **γ**_y_ that link ***Y***_*i*_ to ***X***_*i*_: *f*(***Y***_*i*_| ***X***_*i*_, **γ**_y_). Primary interest generally lies in the inference about the elements contained in **γ**_y_. In a longitudinal study, it is common for measures of the outcome variable to have patterns of incomplete data that vary between individuals, and so a separate model for non-response can be specified to clarify those patterns. Let ***R***_*i*_ = (*R*_1*i*_, …, *R*_*ni*_)^′^ be a set of variables for individual *i* that indicates missingness in the outcome variable at each occasion *t*, *t =* 1, …, *n*, where *R*_*ti*_ = 1 if *Y*_*ti*_ is observed, and *R*_*ti*_ = 0 if *Y*_*ti*_ is missing. In a given problem, several factors could affect non-response, including the outcome ***Y***_*i*_ and covariates. Similar to the data model for ***Y***_*i*_, a multivariate density of ***R***_*i*_ is defined: *f*(***R***_*i*_| ***Y***_*i*_, ***X***_*i*_, **γ**_r_), where **γ**_r_ is a set of unknown parameters that links indicators of non-response to the longitudinal response and covariates. Assuming incomplete longitudinal data, let ***Y***_*i*_ now be a full data vector that is comprised of a set of observed values ***Y***_o*i*_ and missing values ***Y***_m*i*_, where the number of missing and complete values can vary between individuals. Taken together, the joint density of ***Y***_*i*_ and ***R***_*i*_ is

1 $$f\left({\boldsymbol{Y}}_i,{\boldsymbol{R}}_i|{\boldsymbol{X}}_i,{\boldsymbol{\gamma}}_{\textrm{y}},{\boldsymbol{\gamma}}_{\textrm{r}}\right).$$

Rubin (1976) provided a framework for three types of missing data mechanisms, namely missing completely at random (MCAR), missing at random (MAR) and missing not at random (MNAR). Under MCAR, the missingness is independent of the observed and missing values of ***Y***_*i*_. Under MAR, the missingness is dependent on ***Y***_o*i*_ but is independent of ***Y***_m*i*_. Under MNAR, the missingness is dependent on ***Y***_m*i*_, whether or not it is dependent on ***Y***_o*i*_. These mechanisms can be understood by factorization of the joint density in (1). To that end, factorizations of the joint density based on three major modelling frameworks for missing data are reviewed first before returning to add further clarification to the three missing data mechanisms.

## Modelling frameworks for missing data

Three major modelling frameworks for missing data are the selection model, pattern-mixture model and shared parameter model, each distinguished by their factorization of the joint density in (1). For the selection model (cf. Heckman, 1976),

$$f\left({\boldsymbol{Y}}_i,{\boldsymbol{R}}_i|{\boldsymbol{X}}_i,{\boldsymbol{\gamma}}_{\textrm{y}},{\boldsymbol{\gamma}}_{\textrm{r}}\right)=f\left({\boldsymbol{Y}}_i|{\boldsymbol{X}}_i,{\boldsymbol{\gamma}}_{\textrm{y}}\right)f\left({\boldsymbol{R}}_i|{\boldsymbol{Y}}_i,{\boldsymbol{X}}_i,{\boldsymbol{\gamma}}_{\textrm{r}}\right),$$

where the first factor is the marginal density of the longitudinal process that depends on covariates, and the second is the marginal density of the missingness process that is conditional on the longitudinal response and covariates. For the pattern-mixture model (cf. Little, 1993, 1994, Little, 1995),

$$f\left({\boldsymbol{Y}}_i,{\boldsymbol{R}}_i|{\boldsymbol{X}}_i,{\boldsymbol{\gamma}}_{\textrm{y}},{\boldsymbol{\gamma}}_{\textrm{r}}\right)=f\left({\boldsymbol{Y}}_i|{\boldsymbol{R}}_i,{\boldsymbol{X}}_i,{\boldsymbol{\gamma}}_{\textrm{y}}\right)f\left({\boldsymbol{R}}_i|{\boldsymbol{X}}_i,{\boldsymbol{\gamma}}_{\textrm{r}}\right),$$

where the first factor specifies that the marginal density of the longitudinal outcome depends on indicators of missingness and covariates. The second factor specifies that the missingness depends on covariates but not on the longitudinal response. For the shared-parameter model (cf. Wu & Carroll, 1988; Wu & Bailey, 1988, 1989),

$$f\left({\boldsymbol{Y}}_i,{\boldsymbol{R}}_i|{\boldsymbol{X}}_i,{\boldsymbol{\gamma}}_{\textrm{y}},{\boldsymbol{\gamma}}_{\textrm{r}},{\boldsymbol{b}}_i\right)=f\left({\boldsymbol{Y}}_i|{\boldsymbol{X}}_i,{\boldsymbol{\gamma}}_{\textrm{y}},{\boldsymbol{b}}_i\right)f\left({\boldsymbol{R}}_i|{\boldsymbol{Y}}_i,{\boldsymbol{X}}_i,{\boldsymbol{\gamma}}_{\textrm{r}},{\boldsymbol{b}}_i\right),$$

where the first factor is the marginal density of ***Y***_*i*_ that depends on ***X***_*i*_ and random effect ***b***_*i*_, and the second is the marginal density of the missingness process that is conditional on ***Y***_*i*_, ***X***_*i*_ and random effect ***b***_*i*_. Random effects contained in ***b***_*i*_ vary by individual, such as a random intercept or random slope of a linear growth model for ***Y***_*i*_. Clearly, the factorization of the shared parameter model is based on the selection model with the addition that both factors share the random effect ***b***_*i*_. For example, a data model based on a random-effects growth model that includes a random intercept and random slope could specify that the missingness also depends on these two random effects.

Returning to Rubin’s (1976) framework for the three missing data mechanisms, the mechanisms can be clarified using the selection model framework, though it is noted that the missing data mechanisms are not dependent on a particular framework. Under MCAR, the joint density of ***Y***_*i*_ and ***R***_*i*_ can be specified as

$$f\left({\boldsymbol{Y}}_i,{\boldsymbol{R}}_i|{\boldsymbol{X}}_i,{\boldsymbol{\gamma}}_{\textrm{y}},{\boldsymbol{\gamma}}_{\textrm{r}}\right)=f\left({\boldsymbol{Y}}_i|{\boldsymbol{X}}_i,{\boldsymbol{\gamma}}_{\textrm{y}}\right)f\left({\boldsymbol{R}}_i|{\boldsymbol{X}}_i,{\boldsymbol{\gamma}}_{\textrm{r}}\right).$$

The implication of MCAR is that valid inference from the data model can be made independent of the missing data process. Under MAR, the full data vector ***Y***_*i*_ is partitioned into its observed ***Y***_o*i*_ and missing ***Y***_m*i*_ components, and then the joint density is

$$f\left({\boldsymbol{Y}}_i,{\boldsymbol{R}}_i|{\boldsymbol{X}}_i,{\boldsymbol{\gamma}}_{\textrm{y}},{\boldsymbol{\gamma}}_{\textrm{r}}\right)=f\left({\boldsymbol{Y}}_i|{\boldsymbol{X}}_i,{\boldsymbol{\gamma}}_{\textrm{y}}\right)f\left({\boldsymbol{R}}_i|{\boldsymbol{Y}}_{\textrm{o}i},{\boldsymbol{X}}_i,{\boldsymbol{\gamma}}_{\textrm{r}}\right),$$

such that the missing data process depends on observed values of the outcome variable but not those that are missing. The implication of MAR is that valid inference of the longitudinal process can be made independent of the missing data process provided that all available data are analysed. Finally, under MNAR, the joint density is

$$f\left({\boldsymbol{Y}}_i,{\boldsymbol{R}}_i|{\boldsymbol{X}}_i,{\boldsymbol{\gamma}}_{\textrm{y}},{\boldsymbol{\gamma}}_{\textrm{r}}\right)=f\left({\boldsymbol{Y}}_i|{\boldsymbol{X}}_i,{\boldsymbol{\gamma}}_{\textrm{y}}\right)f\left({\boldsymbol{R}}_i|{\boldsymbol{Y}}_i,{\boldsymbol{X}}_i,{\boldsymbol{\gamma}}_{\textrm{r}}\right),$$

such that the missing data process depends on the full data vector that includes observed and missing values of the outcome variable. The implication of MNAR is that valid inference of the longitudinal process cannot be made independent of the missing data process. Herein lies the problem of MNAR in that the observed data alone are not enough to inform the analyst about the missingness. It is therefore up to the analyst to carefully consider possible mechanisms that may account for missing data in a given problem and to then proceed with an understanding that inferences under these assumptions depend on the observed, and not the missing, data.

## Pattern-mixture models

Among the major frameworks for addressing nonignorable missingness in longitudinal data is a random-effects pattern-mixture model in which one or more between-subject indicator variables are created to represent fixed patterns of missing data and individuals are classified according to these patterns (Hedeker & Gibbons, 1997; Little, 1995; Molenberghs & Kenward, 2007). Under the pattern-mixture model factorization of the joint density between the outcome variable and indicators of missingness, the longitudinal outcome is dependent on the missingness. Thus, under this framework, the longitudinal response is conditioned on patterns that describe when the longitudinal outcome is observed and when it is missing. Specifically, between-subject indicator variables are created to represent patterns of missing data. For example, an indicator variable could represent whether or not a subject has complete data versus any pattern of missing data. In a random-effects pattern-mixture model, pattern effects are fixed. Fixed pattern effects are included in the model for the longitudinal response with the assumption that, conditional on the pattern, the missingness is ignorable. Pattern indicators can also be used to predict coefficients of a growth model, such as a random slope, similar to how other subject-level attributes are used to predict characteristics of change in a longitudinal response. Again, conditional on the pattern of missing data, the missingness is assumed to be ignorable. Random-effects pattern-mixture models are generally straightforward to estimate using computer software designed to estimate random-effects models and have been shown to be effective in addressing nonignorable missing data in longitudinal research (Fitzmaurice et al., 2008; Molenberghs & Kenward, 2007).

The random-effects pattern-mixture model is widely applied in the behavioural sciences, possibly due to its ease of application. For instance, in a review of articles that cited Hedeker and Gibbons’ (1997) article on applications of this model, about 200 peer-reviewed articles reported an application of a random-effects pattern-mixture to address data that were possibly MNAR. Of those articles, nearly all applied a single model that represented the missingness by a single variable that denoted each subject’s completion status (e.g., a binary indicator of whether or not a study participant completed the planned assessments or a variable equal to the number of assessments completed), and no alternative models to address the missing data were considered. This is counter, however, to recommendations that researchers consider multiple models that represent plausible explanations about the missing data. Thus, the purpose of this paper is to consider extensions of the random-effects pattern-mixture model and illustrate their applications in assessing the impact of missing data on the statistical inference of a random-effects model for longitudinal data.

## Illness severity in a sample of patients with schizophrenia

Prior to describing models aimed to address nonignorable missingness, a longitudinal study is described that motivates a practice of relying on multiple models that differ in their assumptions about a missing data process and use of a sensitivity analysis to assess the impact of nonignorable missingness under different mechanisms. The data are first analysed by fitting different growth models assuming ignorable missingness. Using the best-fitting model among those considered, models that make different assumptions about the missing data process are applied and compared.

The longitudinal study was designed to examine the effects of psychiatric medications in the treatment of mental illness in a sample of patients with schizophrenia. The National Institute of Mental Health Schizophrenia Collaborative Study was a nationwide controlled study of a psychopharmacological treatment (phenothiazine treatment) in acute schizophrenia (National Institute of Mental Health, Psychopharmacology Service Center Collaborative Study Group, 1964; National Institute of Mental Health, Psychopharmacology Research Branch Collaborative Study Group, 1967). Data were collected from nine public, private and university hospitals. Within hospitals, newly admitted patients diagnosed with schizophrenia and who met the study criteria were randomly assigned to one of four study medications, including a placebo, using a double-blind assignment process. Patients were first stratified according to sex, and within each sex, were randomly assigned to a drug treatment.

**Fig. 1:**
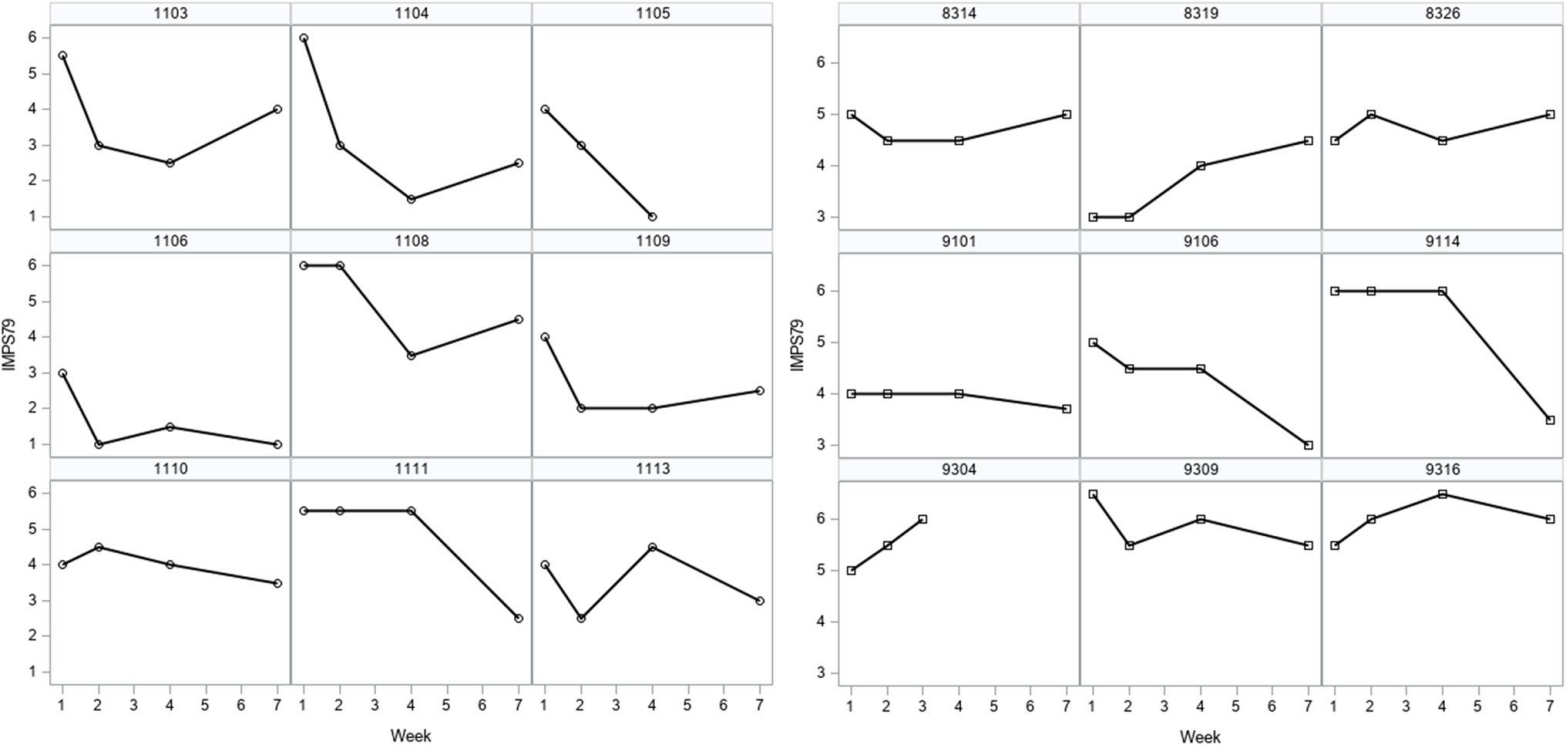
IMPS79 Scores for subsamples of nine patients by group (left: drug; right: placebo)

Data for the 437 patients reported in Fitzmaurice et al. (2011) and Hedeker and Gibbons (1997) are studied here. A global rating of illness severity was measured by Item 79 of the Inpatient Multidimensional Psychiatric Scale (IMPS) (Lorr & Klett, 1966) and assessed using a 7-point ordinal scale. The response scale had values denoting the severity of illness as: 1 = normal, not at all ill; 2 = borderline mentally ill; 3 = mildly ill; 4 = moderately ill; 5 = markedly ill; 6 = severely ill and 7 = among the most extremely ill. Observed ratings, which include non-integer values falling between values of the measurement scale, were obtained beginning with a baseline assessment and follow-ups spanning up to 6 weeks thereafter. Patients who received any psychiatric drug were combined into one group because there were no detectable differences in illness ratings between these groups (see Hedeker & Gibbons). The score is analysed here as a continuous variable. Table 1 provides sample descriptives of illness scores, separately for patients assigned to receive the placebo or a drug, for week = baseline, 1, 2,…,6. Figure 1 displays observed trajectories of nine individuals from each of the two patient groups. Displays of all cases are in Hedeker and Gibbons (1997). Individual differences in the observed trajectories are evident, as is the nonlinearity in the form of change for some patients.

**Table 1:** Descriptive statistics for mental illness scores (n = 437)

	Week	Proportion with missing data	Illness severity score			
			Mean	SD	Minimum	Maximum
Placebo	Baseline	.01	5.4	.83	3	7
	Week 1	.03	5.0	1.2	1	7
	Week 2	.95	5.8	.57	5	6.5
	Week 3	.19	4.7	1.2	1.5	6.5
	Week 4	.98	5.5	.71	5	6
	Week 5	.98	4.3	2.5	2.5	6
	Week 6	.35	4.2	1.4	1.5	6.5
Drug						
	Baseline	.01	5.4	.88	2	7
	Week 1	.02	4.4	1.2	1	7
	Week 2	.97	3.3	1.6	1	6
	Week 3	.13	3.8	1.4	1	7
	Week 4	.97	2.5	1.3	1	5
	Week 5	.98	2.9	1.6	1.5	6
	Week 6	.19	3.1	1.4	1	7

## Patterns of missing data

According to the study’s description, the plan was to obtain illness measures at baseline and 1, 3 and 6 weeks following baseline. For a small number of patients (2–5% of the total subject count), scores were also obtained at weeks 2, 4 and 5. Given the data collection plan, it seemed reasonable to assume that missing scores at weeks 2, 4 and 5 for the vast majority of patients were missing by design, and thus, missing completely at random. Data missing by design are missing completely at random if the missingness is independent of both the observed and the missing data (Graham et al., 2006). Assuming missingness in weeks 2, 4 and 5 is ignorable, the analysis proceeds here in addressing patterns of missing data with regard to baseline and weeks 1, 3 and 6, noting that data from all weeks, as available, are included in the reported analyses.

Indicators of patterns of missing data were generated based on data at baseline and weeks 1, 3 and 6, resulting in nine patterns (see Table 2). Pattern 1 reflects complete data at baseline and weeks 1, 3 and 6 and so corresponds to a pattern assumed to reflect ignorable missingness, as discussed earlier. Patterns 2, 3 and 7 correspond to patterns of monotonic dropout. Patterns 4, 5, 6 and 8 show intermittently missing data. Pattern 9 has a pattern of both intermittently missing data and possible attrition. Fisher’s exact test of independence between pattern and treatment group was statistically significant at the .05 level (*p* = .016). Based on the observed and expected cell counts provided in Table 2, the placebo group has lower than expected counts of patients with complete data at baseline and weeks 1, 3 and 6, whereas the drug group tends to have lower than expected patient counts for patterns involving missing data. Based on this, it may be important to address the missing data when studying differences in illness ratings between the patient groups.

**Table 2:** Patterns of missing data (n = 437)

Week				Pattern	Frequency (expected count)		Total
Baseline	1	3	6		Placebo	Drug	
X	X	X	X	1	64 (77)	248 (235)	312
X	X	X	.	2	19 (13)	34 (40)	53
X	X	.	.	3	18 (11)	27 (34)	45
X	X	.	X	4	3 (3)	10 (10)	13
X	.	X	X	5	2 (1)	3 (4)	5
.	X	X	X	6	1 (1)	2 (2)	3
X	.	.	.	7	0 (1)	3 (2)	3
X	.	.	X	8	0 (0)	2 (2)	2
X	.	X	.	9	1 (0)	0 (1)	1

### Growth models assuming ignorable missingness

Different growth models were first fit to the illness ratings under the assumption that the missingness was ignorable with the goal of characterizing scores across weeks for the two patient groups. Let *y*_*ti*_ denote an illness rating at week *t* for patient *i*. Week was centred to the baseline assessment: week = 0, 1, …, 6. A drug treatment indicator denoted whether or not a patient received one of four psychiatric medications (*Drug*_*i*_ = 0 if patient *i* was given a placebo; *Drug*_*i*_ = 1 if a patient was given a psychiatric drug) and was used to predict each coefficient of a given growth model at the subject level. Illness ratings were assumed to follow a random-effects model:

- Level 1: *y*_*ti*_ = *f*(**β**_*i*_, *week*_*ti*_) + *e*_*ti*_
- Level 2: **β**_*i*_ ***= g***(***γ***, *Drug*_*i*_) ***+ u***_*i*_

where, at the first level, *f*(∙) denotes a function of a set of random coefficients in **β**_*i*_ and the week of assessment, and *e*_*ti*_ is the residual. At the second level, ***g***(∙) denotes a vector-valued function in which the random coefficients at the first level are regressed on the treatment indicator, *Drug*_*i*_, where ***γ*** is a set of fixed effects that link the random coefficients and the treatment indicator; ***u***_*i*_ is the set of residuals corresponding to the level-2 regressions. The conditional random effects ***u***_*i*_ were assumed to be bivariate normal with means equal to zero and variance-covariance matrix **Φ**_*u*_:

$${\boldsymbol{\Phi}}_u=\left[\begin{array}{cc}{\phi}_{u_0}& \\ {}{\phi}_{u_1{u}_0}& {\phi}_{u_1}\end{array}\right]$$

Conditional on the treatment effects, the missingness was assumed to be ignorable.

Four different growth functions (see Table 3) were fit to model change in ratings: linear growth, quadratic growth, linear growth using a square root transformation of week, and exponential growth, with all but the linear model used to address possible nonlinearity in the form of change. The quadratic function allows for non-constant change rates, but due to the parabolic shape of the function, scores would be expected to decrease and later increase with time. The linear function that uses the square root of week changes the time metric and helps to linearize the form of the relationship between the outcome and time. The exponential function includes a lower asymptote to capture stability in illness ratings if it is expected that patients would initially show improvement by a decreasing trend in their clinical ratings and later achieve stability in their illness status.

**Table 3:** Four growth models fitted to illness ratings (n = 437)

Growth form	Level 1	q	Min(ESS)	DIC
Linear	*β*_0*i*_ + *β*_1*i*_*week*_*ti*_	7	8769	4378.320
Linear with $\sqrt{week}$	${\beta}_{0i}+{\beta}_{1i}\sqrt{week_{ti}}$	7	8769	4146.871
Quadratic	${\beta}_{0i}+{\beta}_{1i}{week}_{ti}+{\beta}_{2i}{week}_{ti}^2$	8	8804	4288.535
Exponential	*β*_1*i*_ − (*β*_1*i*_ − *β*_0*i*_) *exp* {−*β*_2*i*_*week*_*ti*_}	8	8804	4204.464
Level 2 regressions				
*β*_0*i*_ = *γ*_00_ + *γ*_01_*Drug*_*i*_ + *u*_0*i*_				
*β*_1*i*_ = *γ*_10_ + *γ*_11_*Drug*_*i*_ + *u*_1*i*_				
$${\beta}_{2i}^{\ast }={\gamma}_{20}+{\gamma}_{21}{Drug}_i$$				

*Notes*: q is the total number of model parameters. “Min(ESS)” is the lower bound on effective sample size calculated using the R package mcmcse (Flegal et al., 2021). DIC is the deviance information criteria. In each function, ‘week’ is centred to the baseline occasion: week = 0,1…,6. The random coefficients of each model were regressed on *Drug* at the second level. The residuals of the first two level-2 equations (e.g., *u*_0*i*_ in the level 2 regression of a model’s intercept) could covary with each other. ${\beta}_{2i}^{\ast }$ was assumed to be non-randomly varying. The residual variance at level 1 could differ between patient groups in each model fitted: ${\sigma}_{ei}^2=\mathit{\exp}\left\{{\tau}_0+{\tau}_1{Drug}_i\right\}.$

To help decipher the information in Table 3, the linear growth model is described as an example. At level 1, the growth function is *β*_0*i*_ + *β*_1*i*_*week*_*ti*_, and at level 2, each growth coefficient is regressed on *Drug*: *β*_0*i*_ = *γ*_00_ + *γ*_01_*Drug*_*i*_ + *u*_0*i*_ and *β*_1*i*_ = *γ*_10_ + *γ*_11_*Drug*_*i*_ + *u*_1*i*_. The intercept of each level 2 regression is the fixed growth coefficient (intercept and slope, respectively) for the placebo group, and the effect of *Drug* is the difference in the growth coefficient between the placebo and drug group. For example, *γ*_00_ is the expected illness rating for the placebo group at baseline, and *γ*_01_ is the expected baseline difference in ratings between the placebo and drug group. The residual of each level 2 regression is the random effect conditional on *Drug.* For example, *u*_0*i*_ is the residual corresponding to the subject-specific intercept of the growth model after conditioning on *Drug.* Although not shown in Table 2, the level 1 residual variance was modelled using an exponential function to permit heterogeneity of variance between treatment groups: ${\sigma}_{e_i}^2=\exp \left\{{\tau}_0+{\tau}_1{Drug}_i\right\}$.

## Estimation

Maximum likelihood and Bayesian estimation are the major approaches to the estimation of random-effects models. Here, estimation is carried out using PROC MCMC for SAS/STAT software version 9.4^1^. Bayesian estimation was selected for the current analysis due to the greater flexibility in how a random-effects model may be specified, a feature that will become evident when models for nonignorable missingness are described. PROC MCMC is a flexible simulation procedure for which Bayesian estimation is carried out by repeatedly sampling from a posterior distribution using the Markov chain Monte Carlo (MCMC) approach (for details, see Gelfand et al., 1990). The primary sampling mechanism for PROC MCMC is a self-tuned random walk Metropolis algorithm (Chen, 2009). The samples drawn are used to estimate the posterior marginal distributions from which statistical inference of the model parameters may be drawn. In fitting a random-effects model using Bayesian methods, the fixed effects and the random effects are treated as random variables.

Weakly informative prior distributions were specified for most of the model parameters. Fixed effects were assumed to have Gaussian priors with mean = 0 and variance = 1000. The prior distribution of the intercept of the growth model was restricted to have a lower bound of 1 and an upper bound of 7, given that the illness rating scale was bounded between 1 and 7. The prior of the variance-covariance matrix of the random coefficients at the subject level and those at the pattern level (when applicable) was assumed to be an inverse-Wishart with small degrees of freedom (e.g., quadratic model assumed 3 df). As an exponential model was used for the residual variance at the first level, the parameters of the variance model were assumed to have Gaussian priors with mean = 0 and variance = 1000. A lower bound for effective sample size (ESS) was calculated using the R package mcmcse (Flegal et al., 2021) assuming a 95% confidence level. The observed ESS of each model parameter was compared to this minimum criterion value. Markov chains were run for 10,000,000 iterations with 50,000 burn-in iterations and thinning set to 1000, with thinning done to reduce memory requirements. Specifications were set to meet the minimum ESS needed across the planned models. Model convergence was judged by inspecting trace and autocorrelation plots and meeting the lower bound ESS for a given model. The posterior mean of fixed effects (assuming symmetric posterior distributions) and the posterior mode for variance parameters (assuming non-symmetric posterior distribution), along with 95% highest posterior density intervals (HPDI), for parameter estimates are reported.

## Results: Growth models assuming ignorable missingness

Indices of fit for the four growth models are given in Table 3. The models take into account possible differences between the treatment groups by including the effects of Drug and assume that the missingness is ignorable. Conditioning on the effects of Drug, the linear model using a square-root transformation of week had the lowest deviance information criterion^2^ (DIC) value. Assuming that a square-root transformation of week provided a more suitable representation than the exponential growth model that included a lower asymptote could be an indication that illness ratings, across the patient samples, were not tending towards clinical stabilization. Going forward, the illness ratings are described by the linear growth model using the square root transformation of week.

Estimates from models assuming ignorable missingness are summarized in the first column of estimates in Table 4. Posterior means of fixed effects and posterior medians for variance parameters, with 95% HPDI, are reported. Based on the estimates, patient groups differed in the expected change over time (${\hat{\alpha}}_{11}$=−0.65, 95% HPDI: (−0.81,−0.49)) but not in their expected baseline levels (${\hat{\alpha}}_{01}$ =0.05, 95% HPDI: (−0.14,0.27)). The residual variance of illness ratings was greater for those assigned to a drug relative to those assigned to the placebo (${\hat{\tau}}_1$=0.28, HPDI: (0.07,0.50)). Between-subject heterogeneity of variance in the expected baseline levels (${\hat{\phi}}_{u_0}$=0.47, HPDI: (0.36, 0.59)) and in the slopes (${\hat{\phi}}_{u_1}$=0.32, HPDI: (0.25, 0.39)) is notable.

**Table 4:** Bayesian estimates of a growth model for illness ratings under different missingness mechanisms (n=437)

	Ignorable missingness	Fixed dropout pattern	Fixed dropout pattern after averaging effects across patterns	Random pattern-mixture on Pattern 1	Random pattern-mixture on Pattern 2	Timing of dropout and growth as independent processes	Timing of dropout depends on growth parameters
Parameter	M (95% Int)	M (95% Int)	M (95% Int)	M (95% Int)	M (95% Int)	M (95% Int)	M (95% Int)
Intercept, *γ*_00_	5.34 (5.16,5.51)	5.22 (5.02,5.43)	5.29 (5.12,5.47)	5.32 (5.10,5.53)	5.32 (5.06,5.56)	5.34 (5.16,5.51)	5.35 (5.18,5.52)
Drug, *γ*_01_	0.05 (−0.14,0.27)	0.20 (−0.02,0.46)	0.11 (−0.09,0.32)	0.05 (−0.14,0.24)	0.06 (−0.14,0.24)	0.05 (−0.14,0.27)	0.05 (−0.14,0.25)
Drop, *γ*_02_		0.32(-−0.05,0.68)					
$\sqrt{week}$, *γ*_10_	−0.33 (−0.46,−0.19)	−0.39 (−0.55,−0.36)	−0.33 (−0.48,−0.21)	−0.34 (−0.52,−0.15)	−0.32 (−0.44,0.25)	−0.33 (−0.46,−0.19)	−0.34 (−0.48,−0.21)
$\sqrt{week}$*Drug, *γ*_11_	−0.65 (−0.81,−0.49)	−0.65 (−0.81,−0.49)	−0.69 (−0.85,−0.53)	−0.66 (−0.80,−0.52)	−0.66 (−0.83,−0.51)	−0.65 (−0.81,−0.49)	−0.65 (−0.82,−0.50)
$\sqrt{week}$*Drop, *γ*_12_		0.26(−0.07,0.56)					
WS model for${\sigma}_{e_i}^2$:							
Intercept, *τ*_0_	−0.81 (−1.01,−0.62)	−0.82 (−1.02,−0.63)	−0.82 (−1.02,−0.63)	−0.74 (−0.95,−0.55)	−0.74 (−1.01,−0.61)	−0.81 (−1.01,−0.62)	−0.81 (−1.00,−0.61)
Drug, *τ*_1_	0.28 (0.07,0.50)	0.28 (0.06,0.50)	0.28 (0.06,0.50)	0.28 (0.06,0.49)	0.28 (0.05,0.50)	0.28 (0.07,0.50)	0.28 (0.06,0.50)
BS model:							
${\phi}_{u_0}^2$	0.47 (0.36,0.59)	0.47 (0.36,0.58)	0.47 (0.36,0.58)	0.34 (0.21,0.47)	0.34 (0.36,0.58)	0.47 (0.36,0.59)	0.47 (0.36,0.59)
${\phi}_{u_1{u}_0}$	−0.03 (−0.09,0.04)	−0.04 (−0.10,0.02)	−0.04 (−0.10,0.02)	0.04 (−0.03,0.11)	0.04 (−0.09,0.03)	−0.03 (−0.09,0.04)	−0.03 (−0.09,0.04)
${\phi}_{u_1}^2$	0.32 (0.25,0.39)	0.31 (0.24,0.38)	0.31 (0.24,0.38)	0.22 (0.16,0.29)	0.23 (0.25,0.39)	0.32 (0.25,0.39)	0.32 (0.25,0.39)
Random pattern:							
${\phi}_{v_0}^2$				0.01 (0.00,0.03)	0.02 (0.00,0.05)		
${\phi}_{v_1{v}_0}$				−0.00 (−0.02,0.01)	−0.00 (−0.03,0.02)		
${\phi}_{v_1}^2$				0.01 (0.00,0.03)	0.01 (0.00,0.05)		
Week-of-last-observation model:							
Intercept, *α*_0_						1.63 (1.54,1.72)	1.63 (1.54,1.72)
Drug, *α*_1_						0.13 (0.03,0.24)	0.13 (0.03,0.23)
Random intercept, *α*_2_							−0.01 (−0.10,0.07)
Random slope, *α*_3_							0.09 (−0.05,0.22)
WS model for${\sigma}_{\varepsilon_i}^2$:							
Intercept, *κ*_0_						−1.49 (−1.75,−1.23)	−1.47 (−1.75,−1.18)
Drug, *κ*_1_						−0.31 (−0.64,−0.02)	−0.36 (−0.70,−0.04)

*Note*: M = posterior mean for fixed effects, M = posterior mode for variance parameters. Under the growth model, “WS model” refers to the within-subject residual variance model and “BS model” refers to the between-subject residual variance-covariance model. Under the week-of-last-observation model, “WS model” refers to the within-subject residual variance model.

## Models for nonignorable missingness

As described earlier, about 23.3% of patients are considered to have (unplanned) incomplete data. Reasons for the missing data are not described in the documentation cited previously for the National Institute of Mental Health Schizophrenia Collaborative Study, so it is reasonable to consider different scenarios that might account for the sources of the missing data, and importantly, study how inferences about the longitudinal process are sensitive to different assumptions.

The patterns in Table 2 are used to formulate models that represent possible missing data processes. Three of the nine patterns (patterns 2, 3 and 7) reflect a monotonic dropout pattern, and four others (patterns 4, 5, 6 and 8) reflect intermittently missing data. The last pattern (pattern 9) reflects intermittently missing data but possible attrition near the end of the planned observation period. Based on the result of a Fisher’s exact test of independence between pattern and treatment group, the patterns of missing data may be informative in the analysis of the illness ratings. The models considered here use information from the patterns in multiple ways with a goal of representing multiple plausible models for the missing data. The goal in fitting multiple models that reflect plausible missing data processes was to evaluate if the parameters of the longitudinal model for the illness ratings were sensitive to the assumptions made about the missingness.

The first model considered is a pattern-mixture random-effects model with a single fixed pattern of dropout. This model uses the 6^th^ week of observation to indicate whether or not a patient provided data at the final planned assessment. Thus, this single indicator groups the 101 individuals with patterns of monotonic dropout and the one individual with a combination of intermittently missing data and dropout patterns. This model assumes that patterns of intermittently missing data are not important and that the effects of monotonic dropout, regardless of the timing, do not differ from each other. The second and third models are random pattern-mixture models that treat the missing data pattern as a random effect. Specifically, the second model uses all nine patterns and assumes that monotonic dropout and intermittently missing data patterns are important, and the third model uses five of the nine patterns to include only monotonic dropout patterns.

Subject attrition is common in longitudinal investigations, and so additional models were specified in which the growth model for illness ratings was estimated jointly with a model for the week when the patient was last observed. In the first model, the timing of dropout was regressed on Drug, and in the second model, was additionally regressed on the random intercept and slope of the growth model. Thus, the latter model links the timing of dropout to the illness ratings through the random growth coefficients that characterize change in the illness ratings, and the former assumes the two processes are independent. The second of these two models is known as a shared parameter model in which coefficients of one model are shared with those of the other model and where estimation of the two models is done jointly (Albert & Follman, 2009; Wu & Carroll, 1988).

### Fixed pattern-mixture model

The first model was a random-effects model with a single fixed-pattern effect. An indicator of dropout was assumed to account for differences in the longitudinal trajectories between those who completed treatment and those who did not, defined by whether or not the patient was observed at the 6^th^ week. The indicator, henceforth called Drop, was equal to 1 for patients with patterns *k* = 2, 3, 7 and 9 in Table 2 (n = 102 (23.3%) patients) and otherwise was equal to 0 (see Hedeker & Gibbons, 1997). To fit this model, the model in Eq. (1) was extended to include the effect of Drop and its interaction with Drug:

$${y}_{tik}={\beta}_{0 ik}+{\beta}_{1 ik}\sqrt{week_{tik}}+{e}_{tik,}$$

where

$${\beta}_{0 ik}={\gamma}_{00}+{\gamma}_{01}{Drug}_{ik}+{\gamma}_{02}{Drop}_{ik}+{\gamma}_{03}{Drug}_{ik}\ast {Drop}_{ik}+{u}_{0 ik}$$

$${\beta}_{1 ik}={\gamma}_{10}+{\gamma}_{11}{Drug}_{ik}+{\gamma}_{12}{Drop}_{ik}+{\gamma}_{13}{Drug}_{ik}\ast {Drop}_{ik}+{u}_{1 ik}.$$

The coefficients of the growth model are functions of Drug, Drop and their interaction. The residuals *u*_0*ik*_ and *u*_1*ik*_ of the level 2 equations are conditional random effects. As was done in the model that assumed ignorable missingness and all forthcoming models that assume nonignorable missingness, the two random effects could covary.

The growth coefficients were allowed to differ between groups based on the indicator of dropout. To ease the comparison of estimates from this model to other models, the overall population effects are calculated by averaging across patterns, weighted by the proportions of subjects within patterns (cf. Little, 1993; 1995; Hogan & Laird, 1997):

$$\overline{\gamma}={\pi}^{Drop=0}{\gamma}^{Drop=0}+{\pi}^{Drop=1}{\gamma}^{Drop=1}$$

where *γ* is a fixed growth coefficient, such as the model’s intercept, *π*^*Drop* = 0^ is the population proportion of individuals with no pattern of dropout, and *π*^*Drop* = 1^ is the population proportion of individuals with a pattern of dropout. Using the sample proportion of patients with a pattern of dropout (.2334), estimates of the population averages were obtained for the model’s fixed intercept and the effects of $\sqrt{week}$, *Drug* and the interaction between *Drug* and $\sqrt{week}$.

### Random pattern-mixture model

Next, random pattern-mixture models were specified. Two pattern sets were tested, each set assumed to be from a population of missing data patterns. The first, Pattern Set 1, included patterns of intermittently missing data, patterns of monotonic dropout and a combination of the two (pattern 9). If intermittently missing data were missing at random, and thus, ignorable, then it would not be important to include those patterns in an analysis. So, a second set, Pattern Set 2, included only the monotonic pattern of missingness (patterns 2, 3, 7 and 9; as pattern 9 possibly has a pattern of attrition, it was included here as a pattern of dropout). Note that Pattern Set 2 is comprised of patterns used to make the indicator Dropout, but in this model, the pattern effect is assumed to be random, and as such, the model permits differences in effects due to differences in the timing of when a patient dropped from the study. If a patient was considered to have complete data, then missingness was assumed to be ignorable and their model was specified by Eqs. (1) and (2). Otherwise, patients who had a pattern of missing data had a longitudinal model that included the random patterns effect *v*_0*k*_ and *v*_1*k*_:

$${y}_{tik}={\beta}_{0 ik}+{\beta}_{1 ik}\sqrt{week_{tik}}+{e}_{tik},$$

where, at the subject level,

$${\beta}_{0 ik}={\gamma}_{00k}+{\gamma}_{01}{Drug}_{ik}+{u}_{0 ik}$$

$${\beta}_{1 ik}={\gamma}_{10k}+{\gamma}_{11}{Drug}_{ik}+{u}_{1 ik,}$$

and at the pattern level,

$${\gamma}_{00k}={\gamma}_{00}+{v}_{0k}$$

$${\gamma}_{10k}={\gamma}_{10}+{v}_{1k}.$$

Conditional on a random pattern of missing data, missingness was assumed to be ignorable. The random pattern effects *v*_0*k*_ and *v*_1*k*_ were assumed to be bivariate normal with means equal to zero and variance-covariance matrix **Φ**_*v*_:

$${\boldsymbol{\Phi}}_v=\left[\begin{array}{cc}{\phi}_{v_0}& \\ {}{\phi}_{v_1{v}_0}& {\phi}_{v_1}\end{array}\right].$$

Estimation of the data model for illness ratings was also carried out simultaneously with a model that predicted the log-transformed (to reduce positive skew) value of the week when a patient was last observed, henceforth called ln(MaxWeek). The variable MaxWeek might be considered a proxy for the actual time of dropout from the study. A higher value of ln(MaxWeek) indicates greater time spent in the study. As the models that aim to address nonignorable missingness take into account a patient’s pattern of missingness, the outcome *y*_*tik*_ includes an added subscript *k* to denote the missing pattern for the individual. The joint model is presented as

1 $${y}_{tik}={\beta}_{0 ik}+{\beta}_{1 ik}\sqrt{week_{tik}}+{e}_{tik}$$

where

$${\beta}_{0 ik}={\gamma}_{00}+{\gamma}_{01}{Drug}_{ik}+{u}_{0 ik}$$

$${\beta}_{1 ik}={\gamma}_{10}+{\gamma}_{11}{Drug}_{ik}+{u}_{1 ik,}$$

and

2 $$\ln {(MaxWeek)}_{ik}={\alpha}_0+{\alpha}_1{Drug}_i+{\varepsilon}_{ik}.$$

The model for ln(MaxWeek)_*ik*_ includes an intercept, *α*_0_, a treatment effect, *α*_1_ and the residual of the regression, *ε*_*ik*_. Similar to the residual variance of the model for illness ratings, the residual variance of the model for the last week of observation was allowed to differ between treatment groups by using an exponential model: ${\sigma}_{\varepsilon_i}^2=\exp \left\{{\kappa}_0+{\kappa}_1{Drug}_i\right\}$. Thus, under this model, the longitudinal process and the timing of dropout are assumed to be independent.

### Shared parameter, random-effects model

Last, a shared parameter random-effects model was fit in which the random intercept and slope of the model for illness ratings were shared parameters in the model for ln(*MaxWeek*)_*ik*_:

$${y}_{tik}={\beta}_{0 ik}+{\beta}_{1 ik}\sqrt{week_{tik}}+{e}_{tik}$$

where

$${\beta}_{0 ik}={\gamma}_{00}+{\gamma}_{01}{Drug}_{ik}+{u}_{0 ik}$$

$${\beta}_{1 ik}={\gamma}_{10}+{\gamma}_{11}{Drug}_{ik}+{u}_{1 ik},$$

and

$$\ln {(MaxWeek)}_{ik}={\alpha}_0+{\alpha}_1{Drug}_i+{\alpha}_2{\beta}_{0 ik}+{\alpha}_3{\beta}_{1 ik}+{\varepsilon}_{ik},$$

where *α*_2_ and *α*_3_ are the effects of the random intercept *β*_0*ik*_ and slope *β*_1*ik*_ of the longitudinal model on the timing of dropout. Under this model, it is assumed that the timing of dropout is dependent on the subject-specific aspects of the longitudinal trajectory. Thus, nonignorable missingness is accounted for through the relationship between the timing of dropout and aspects of change in the illness ratings that characterize the observed and the missing illness ratings. Conditional on the random coefficients *β*_0*ik*_ and *β*_1*ik*_, the longitudinal response *y*_*tik*_ and the week of the last observation ln(*M*axWeek)_*ik*_ are independent. Finally, the residual in the model for the timing of dropout was assumed to be normally distributed with mean equal to 0 and a variance that could different between treatment groups. Specifically, similar to the model used to represent the residual variance of the growth model for illness ratings, an exponential function was used to model the residual variance for the regression of the timing of dropout: ${\sigma}_{\varepsilon_i}^2=\exp \left\{{\kappa}_0+{\kappa}_1{Drug}_i\right\}$.

## Results

Results from fitting the model that assumed ignorable missingness (described earlier) and those that assumed a mechanism of nonignorable missingness are summarized in Table 4. The posterior mean for fixed effects and the posterior median for variance parameters, with 95% HPDI, are reported for the parameter estimates. For the pattern-mixture model with a fixed pattern effect that reflected whether or not a patient had complete data, estimates are given for the model with estimates based on how the model was parameterized (discussed earlier), as well as a set of estimates for which the fixed effects are the population-averaged estimates, as previously discussed. For the random pattern-mixture models, estimates are provided for the model that used Pattern Set 1 to define the pattern effects in which the effects related to attrition and intermittently missing data, and for the model that used Pattern Set 2 to define the pattern effects in which the effects related only to patterns of attrition. Finally, estimates are provided for the joint model for the longitudinal outcome and the timing of dropout, followed by estimates of the shared parameter model.

From the table of parameter estimates, it is clear that similar conclusions can be drawn about the marginal growth parameters for the two patient populations. That is, whether the missingness is considered to be ignorable or nonignorable, similar conclusions are reached about treatment effects on the illness trajectories of the two patient groups. The pattern-mixture model with a fixed pattern effect showed differences in the expected change between those with complete data versus those with incomplete data. The average effects, however, yield estimated population parameters that are close to the estimates resulting from all other models. This is similar to the results reported in Hedeker and Gibbons (1997). Estimates between the two random pattern-mixture models were comparable whether patterns of intermittently missing data were included or not when defining the random pattern effect, and estimates from these two models were comparable to those from all other models that were fit. Under the shared parameter model in which timing of dropout was regressed on the random intercept and slope of the longitudinal model, dropout was not dependent on the random coefficients of the growth model, a result that also suggests ignorable missingness.

## A simulation study

To validate the random pattern-mixture model as a viable approach to addressing non-ignorable missingness, a small simulation study was conducted. A set of 100 data sets was generated for 400 subjects, measured from one to up to six occasions (coded as *wave =* 1,…6) under a random-effects linear growth model for a single normal variable *y* with a subject-level binary covariate *X*_1*i*_ (simulated as *X*_1*i*_~*N*(0, 1) with a cutpoint at 0) and a subject-level continuous covariate *X*_2*i*_ (*X*_2*i*_~*N*(0, 1)). Unlike the covariate *X*_1*i*_ that was used in the generating model and the models fitted for analysis, *X*_2*i*_ was only used in the data-generating model to simulate an unmeasured covariate that was related to *y*_*ti*_ through the parameters of the growth model, related to the covariate *X*_1*i*_, and predicted missingness in *y*_*ti*_. The response *y*_*ti*_ at wave *t* for subject *i* was generated by

$${y}_{ti}={\beta}_{0i}+{\beta}_{1i}\left({wave}_{ti}-1\right)+{e}_{ti}$$

where

$${\beta}_{0i}={\gamma}_{00}+{\gamma}_{01}{X}_{1i}+{\gamma}_{02}{X}_{2i}+{u}_{0i}$$

$${\beta}_{1i}={\gamma}_{10}+{\gamma}_{11}{X}_{1i}+{\gamma}_{12}{X}_{2i}+{u}_{1i},$$

where *γ*_00_ = 1, *γ*_01_ = 0.5, *γ*_02_ = 1, *γ*_10_ = 2, *γ*_11_ = 0.2 and *γ*_12_ = 0.5. Further, *X*_1*i*_ = .5*X*_2*i*_ + *e*_*xi*_.

The residual at the first level was assumed to be independent and identically distributed (i.i.d.) normal as ${\boldsymbol{e}}_i\sim \left(\textbf{0},{\sigma}_e^2{\textbf{I}}_{\textbf{6}}\right)$, where ${\sigma}_e^2=0.3$ and **I**_**6**_ was a 6 × 6 identity matrix. The residuals at the second level were assumed to be independent between subjects and multivariate normal:

$$\left[\begin{array}{c}{u}_{0i}\\ {}{u}_{1i}\end{array}\right]\sim mvn\left(\left[\begin{array}{c}0\\ {}0\end{array}\right],\left[\begin{array}{cc}{\phi}_{u_0}& \\ {}{\phi}_{u_1{u}_0}& {\phi}_{u_1}\end{array}\right]\right),$$

where $${\phi}_{u_0}=1$$, $${\phi}_{u_1}=0.5$$, and $${\phi}_{u_1{u}_0}=0.1$$.

Missingness in *y*_*ti*_ was generated according to a logistic regression model for a set of binary dependent variables that represented missing (*R*_*ti*_ = 1) or not missing (*R*_*ti*_ = 0) in *y*_*ti*_ at waves *t* = 2,…,6, where missingness depended on the covariates *X*_1*i*_ and *X*_2*i*_. Letting *η*_*t*_ denote the logit at wave *t* of the probability that *y*_*ti*_ was missing, *η*_*t*_ by wave was specified as

$${\eta}_2=-1+0.2{X}_{1i}+0.3{X}_{2i}$$

$${\eta}_3=-1+0.4{X}_{1i}+0.6{X}_{2i}$$

$${\eta}_4=-1+0.8{X}_{1i}+1.2{X}_{2i}$$

$${\eta}_5=-1+1.6{X}_{1i}+2.4{X}_{2i}$$

$${\eta}_6=-1+3.2{X}_{1i}+4.8{X}_{2i}.$$

The probability that *y*_*ti*_ was missing when *X*_1*i*_ = 0 and *X*_2*i*_ = 0 was *P*(1/(1 + exp {1})) = .27, with the probability of missingness increasing over waves for *X*_1*i*_ = 1 and increased values of *X*_2*i*_ according to the coefficients specified in the equations relating to the logit. This data-generating model therefore generated both monotonic and non-monotonic missingness in *y*_*ti*_.

Four linear growth models, with *X*_1*i*_ as a predictor of the random intercept and slope, were fitted to the simulated data. The first assumed that the missingness was ignorable. The second model included an indicator variable that denoted whether or not an individual had any pattern of monotonic dropout (*drop*_*i*_= 1; *drop*_*i*_= 0 otherwise). Thus, this model ignored patterns of intermittently missing data and assumed that the effects of monotonic missingness were equal. The third model included a numeric covariate that denoted the timing of monotonic dropout (*timing*_*i*_), with this model also ignoring patterns of intermittently missing data. The fourth model clustered subjects by pattern of missing data. For each parameter, the average 95% credible interval is reported along with bias in Table 5. As shown in Table 5, the magnitude of parameter bias is consistently lowest under the random pattern-mixture model that captures patterns of monotonic and non-monotonic missingness in *y*_*ti*_. Though none of the fitted models was the model that generated the data, the random pattern-mixture model provided parameter estimates of the growth model that were closest to the true parameter values.

**Table 5:** Results from 100 simulated data sets under different assumed missingness data mechanisms (n=400 subjects)

		Ignorable missingness		Single dropout pattern		Timing of dropout		Random pattern-mixture	
Parameter	True value	Bias	AIW	Bias	AIW	Bias	AIW	Bias	AIW
Intercept, *γ*_00_	1	−0.24	0.76	−0.42	0.58	−0.37	0.63	0.05	1.05
*X*_1_, *γ*_01_	0.5	0.48	0.98	0.20	0.70	0.34	0.84	−0.18	0.32
*X*_2_, *γ*_02_	1								
drop,					1.05				
timing							0.22		
wave, *γ*_10_	2	−0.14	1.86	−0.22	1.78	−0.19	1.81	0.02	2.02
*X*_1_ ∗ *wave*, *γ*_11_	0.2	0.22	0.42	0.11	0.31	0.16	0.36	−0.10	0.10
*X*_2_ ∗ *wave*, *γ*_12_	0.5								
*drop* ∗ *wave*					0.49				
*timing* ∗ *wave*							0.11		
WS model for ${\sigma}_{e_i}^2$:									
Intercept, *τ*_0_	−1.20	−0.01	−1.21	−0.01	−1.21	−0.01	−1.21	−0.01	−1.21
BS model:									
${\phi}_{u_0}^2$	1	0.94	1.94	0.73	1.73	0.84	1.84	0.36	1.36
${\phi}_{u_1{u}_0}$	0.1	0.45	0.55	0.36	0.46	0.40	0.50	0.18	0.28
${\phi}_{u_1}^2$	0.5	0.23	0.73	0.19	0.69	0.20	0.70	0.09	0.59
Random pattern:									
${\phi}_{v_0}^2$									0.15
${\phi}_{v_1{v}_0}$									0.07
${\phi}_{v_1}^2$									0.04

*Note*: Bias = AIW [average interval width] − true value, where AIW = average 95% credible interval. “WS model” refers to the within-subject residual variance model and “BS model” refers to the between-subject residual variance-covariance model. Bias is not applicable to the effects of ‘drop’ or ‘timing’, as well as the estimated three-level variances and covariance of the random pattern effects, because these parameters were not part of the data-generating model.

## Discussion

Missing data in longitudinal studies are common, making it important in many problems for the analysis to address reasons why data are missing, and importantly, how they may impact inference from a longitudinal model. The issue is that the data from subjects with missing data may be different from those of subjects with complete data. If that is the case, then inference from a longitudinal model that assumes ignorable missingness will not reflect the full population that would include a combination of subjects with either complete or incomplete data. If data are missing solely by design (Graham et al., 2006), then it is reasonable to assume that the missingness is ignorable, leaving no need to also account for the missingness in the analysis. If data show patterns of subject attrition, however, then it is advisable to consider different scenarios about the source of the missingness. If correlates of the missingness or the missing data are available, then such variables can be included in the analysis, such as by including correlates as covariates. In other situations where correlates of the missingness or the missing values are not available, then the researcher may consider models that reflect nonignorable missingness, including the use of pattern-mixture, selection models and shared parameter models. This may be done by specifying a pattern-mixture model with one or more fixed pattern effects that allow the marginal effects of a growth model to differ according to groups defined by a finite number of missing data patterns (Hedeker & Gibbons, 1997). Alternatively, the pattern effect may be random (Guo et al., 2004), an option that may be more suitable to problems involving many patterns of missing data, including patterns reflective of intermittently missing data and subject attrition. Another option is to specify a shared parameter model in which the missingness depends on the observed and missing values of the measured outcome (Albert & Follman, 2009; Wu & Carroll, 1988). Here, for example, the missingness was represented by the last week that a patient was observed, and the observed and missing values of the measured illness ratings were presented by the random coefficients of the growth model.

Using a set of empirical data from a longitudinal study, this paper illustrated these major frameworks for dealing with nonignorable missingness. The aim was to show how to compare estimates of a longitudinal model under a range of different possible mechanisms of the missing data to assess whether inference from the model was sensitive to the assumptions made about the missing data (Daniels & Hogan, 2008). A pattern-mixture model was applied in which the pattern of missing data was fixed in one version of the model and assumed to be random in a different version. The version in which the pattern was fixed is the same as one of the models reported in Hedeker and Gibbons (1997). In the version of a pattern-mixture model that assumed a random pattern effect, it was possible to account for patterns of intermittently missing data and patterns of attrition. Although a random pattern-mixture model has been previously considered by Guo et al. (2004) (for a different set of empirical data), their application of the model was one in which the random pattern was designed to capture the effects of subject attrition. The application here proposes use of a random pattern-mixture model as a tool for evaluating whether intermittently missing data are possibly nonignorable.

In addition to fitting a pattern-mixture model with either a fixed or random pattern effect, a shared parameter model was applied in which the timing of dropout was dependent on the random coefficients of the longitudinal model in what is called a shared parameter model. A shared parameter model in the context of missing data is a special case of a class of models known as selection models in which missingness is predicted by the observed and the missing values of the measured response of the growth models (Albert & Follman, 2009; Wu & Carroll, 1988). Here, the link between missingness and the illness ratings was made through a variable representing the last week a patient was observed and the random effects of the growth model that represented the illness ratings. Thus, in this nonignorable model, the missingness was specified to be related to both the observed and the missing values of the primary response through the random coefficients of the growth model. In a selection model, the assumption is that the parameters of the longitudinal response and dropout are independent after conditioning each on the random effects of a growth model.

Comparisons were made between the estimated fixed effects of the growth model, and inference about the marginal growth parameters did not differ greatly between models. Under the random pattern-mixture model in particular, it did not matter whether the random pattern effect included patterns of attrition alone or a combination of patterns reflecting attrition and intermittently missing data. Thus, including additional patterns to reflect intermittently missing data did not result in a different conclusion about group-level change in the illness ratings. Inference from the marginal longitudinal model also did not differ if dropout was allowed to depend on the random effects of the growth model. Thus, conditioning on the drug treatment effects, the missingness is arguably ignorable for the marginal aspects of illness ratings. This is consistent with the conclusions about the missingness for this particular data set that was presented in Hedeker and Gibbons (1997). (For examples of a sensitivity analysis that does result in differences between models, please see Molenberghs & Kenward, 2007).

The small collection of models considered here are used as a means to model nonignorable missingness in applications of random-effects models for longitudinal data. The work here relied on Bayesian estimation, instead of maximum likelihood estimation that is more common. Bayesian estimation was used primarily because this approach provides a great deal of flexibility in an analysis, which seemed particularly applicable to the estimation of the pattern-mixture model that assumed a random pattern effect. If the number of patterns of missing data is small, then using Bayesian estimation can permit testing of a model that assumes that the standard deviation of a single random pattern effect follows a half *t* distribution (Chen et al., 2016). This kind of problem is analogous to fitting three-level models for which the number of random subjects at the highest level is small (Gelman, 2006).

The empirical example presented in this paper was used to illustrate different ways in which one might address nonignorable missingness in a longitudinal data analysis that uses a random-effects model. Naturally, there are variations in the specific models that were tested here, such as those considered for a pattern-mixture model with a fixed pattern effect (see Hedeker & Gibbons, 1997). For example, for the illness ratings that were analysed in this report, the patterns of missing data included those reflective of attrition, as well as those reflective of intermittently missing data. Thus, in the analysis of this data set, models for nonignorable missingness were specified to reflect both patterns. Finally, it is also important to mention that although different models may be considered to represent a missing data process, the fact that an analysis may suggest that the missingness is ignorable does not imply certainty in that conclusion. That is, the models that one uses to represent a nonignorable process may not capture the true underlying process. For this reason, researchers must carefully consider including additional variables that may be correlated with either the missingness or the primary variables of a data model. If these added variables are correlated with the missingness or the variables of the data model, then conditioning on their effects may help to account for the missingness (Little & Rubin, 2002).

This paper focused on some of the major frameworks for analysing longitudinal data that are MNAR. These methods represent different ways in which an analyst can model missingness and its possible impact on inference from the substantive model that is often the primary interest. An important shortcoming from the application of any one framework in which an analyst then chooses to model missingness by a single model is that inference from the substantive model is done under the assumption that the model for missingness generated the missing data. Obviously, this strategy ignores the uncertainty about the true source of the missing data, in part from only having observed data available for analysis, but also from considering only a single model to represent the missingness. It is therefore recommended that data be analysed under multiple models of plausible mechanisms of missingness, as was done in this paper, with an understanding of the likely possibility that no one model of those considered accurately captures the missing data process.

One major strategy for handling missing data that was not considered here is multiple imputation (MI). The central idea of MI is to replace missing values in a data set with a set of multiple plausible values from which inference is drawn about the parameters of the marginal model. The process by which data are imputed is not necessarily dependent on the specification of a missingness process, although some approaches to using MI have included aspects of a pattern-mixture model to define the imputation model (e.g., Little & Yau, 1996; Thijs, Molenberghs, Verbeke, & Curran, 2002). A benefit of MI methods is that they can ease the constraints in how the mechanism for missingness is represented in the imputation model, and importantly, permit the inclusion of variables that predict missingness in the imputation process. This is helpful in situations in which there is no interest in including these particular variables as covariates in a longitudinal model. That is, these auxiliary variables can provide valuable information about the missing data during the imputation process but will not interfere with the goals of modelling the longitudinal outcome.

MI is indeed a Bayesian approach to missing data (Schafer, 1997). MI methods have the desirable aspect of being able to include many auxiliary variables to address sources of nonignorable missingness and are naturally not dependent on model specifications regarding particular missing data processes that are inherent to the frameworks considered in this paper. That said, MI also involves uncertainty in the imputation model itself, and methods have been designed to address this (Hinne et al., 2020; Kaplan & Yavuz, 2020) that might also be applied in accounting for nonignorable missingness in the context of longitudinal data.

### Supplementary Information

ESM 1 (CSV 23 kb)

ESM 2 (DOCX 13 kb)

ESM 3 (DOCX 12 kb)

## Data Availability

A dataset and scripts for analyses presented in the study are included as Supplementary Materials.

## Footnotes

1 SAS System for Windows. Copyright © 2016 by SAS Institute Inc. SAS and all other SAS Institute Inc. product or service names are registered trademarks or trademarks of SAS Institute Inc., Cary, NC, USA.

2 The deviance information criterion (Spiegelhalter et al., 2002) is calculated using the posterior mean estimates of the model parameters where both fixed and random effects are treated as random variables; this is in contrast to a marginal information criterion that is conditional on the fixed effects alone.
