# Supplementary material for: Bayesian pattern-mixture models for dropout and intermittently missing data in longitudinal data analysis
Source: Behav Res Methods. 2023 May 23;56(3):1953–67. doi: 10.3758/s13428-023-02128-y (PMC10990982; doi:10.3758/s13428-023-02128-y)
Supplement: Supplementary file 2 — (DOCX 13 kb) [file 13428_2023_2128_MOESM2_ESM.docx]

title1 "ignorable missingness";

title2 "linear growth by sqrt week + heterogeneity of variance by drug";

proc mcmc data=all seed=465798789 nbi=200000 nmc=50000000 thin=500 outpost=outMixed plots=all;

array alp [4] alp00 alp01 alp10 alp11;

array theta[2] b0i b1i; * subject level random effects;

array tau[2] tau0 tau1;

array mu[2];

array Sig[2,2];

array Sig0[2,2];

array S[2,2] (.01 0 0 .01);

begincnst;

call zeromatrix(mu);

call identity(Sig0);

call mult(Sig0,1000,Sig0);

endcnst;

beginnodata; * define a constant for the log-likelihood function;

pi = arcos(-1);

endnodata;

parms alp00 5 alp01 0 alp10 0 alp11 0; * parms is a statement to provide starting values;

parms Sig {0.41 0 0 2.2} tau0 -0.5 tau1 0;

prior alp: ~ normal(0,var=1000); * prior distributions for fixed effects, weakly informative;

prior Sig ~ iwish(2,S); * prior distributions for covariance matrix, inverse Wishart;

prior tau: ~ normal(0,var=1000); * prior distributions for effects that define the within-subject variance;

random theta ~ mvn(mu,Sig) subject=id; * subject-level random intercept and slope with covariance;

f0 = alp00 + alp01*drug + b0i; * random intercept as a function of drug;

f1 = alp10 + alp11*drug + b1i; * random slope as a function of drug;

pred = f0 + f1*sqrt(wk); * predicted value of y;

s2e = exp(tau0+tau1*drug); * exponential level-1 residual variance model as a function of drug;

LL = (-.5)*((((y-pred)**2)/s2e) + log(s2e) + log(2*pi)); * normal log-likelihood;

model y ~ general(LL);

title1 "fixed pattern of dropout";

title2 "linear growth by sqrt week + heterogeneity of variance by drug";

proc mcmc data=all seed=465798789 nbi=200000 nmc=50000000 thin=500 outpost=outMixed plots=all;

array alp [6] alp00 alp01 alp02 alp10 alp11 alp12;

array theta[2] b0i b1i;

array tau[2] tau0 tau1;

array mu[2];

array Sig[2,2];

array Sig0[2,2];

array S[2,2] (.01 0 0 .01);

begincnst;

call zeromatrix(mu);

call identity(Sig0);

call mult(Sig0,1000,Sig0);

endcnst;

beginnodata; * define a constant for the log-likelihood function;

pi = arcos(-1);

endnodata;

parms alp00 5 alp01 0 alp02 0 alp10 0 alp11 0 alp12 0; * parms statement to provide starting values;

parms Sig {0.41 0 0 2.2} tau0 -0.5 tau1 0;

prior alp: ~ normal(0,var=1000); * prior distributions for fixed effects, weakly informative;

prior Sig ~ iwish(2,S); * prior distributions for covariance matrix, inverse Wishart;

prior tau: ~ normal(0,var=1000); * prior distributions for effects that define the within-subject variance;

random theta ~ mvn(mu,Sig) subject=id; * subject-level random intercept and slope with a covariance;

f0 = alp00 + alp01*drug + alp02*drop + b0i; * random intercept as a function of drug and drop;

f1 = alp10 + alp11*drug + alp12*drop + b1i; * random slope as a function of drug and drop;

pred = f0 + f1*sqrt(wk); * predicted value of y;

s2e = exp(tau0+tau1*drug); * exponential level-1 residual variance model as a function of drug;

LL = (-.5)*((((y-pred)**2)/s2e) + log(s2e) + log(2*pi)); * normal log-likelihood;

model y ~ general(LL);

title1 "random pattern";

title2 "linear growth by sqrt week + heterogeneity of variance by drug";

proc mcmc data=all seed=465798789 nbi=200000 nmc=50000000 thin=500 outpost=outMixed plots=all;

array alp [4] alp00 alp01 alp10 alp11;

array theta[2] b0i b1i; * subject level random effects;

array mu[2] (0 0);

array pj[2] pj0 pj1; * pattern-level random effects;

array Sig[2,2];

array S[2,2] (.01 0 0 .01);

array Sig0[2,2];

array S0[2,2] (.01 0 0 .01);

beginnodata;

pi = arcos(-1);

endnodata;

parms alp00 0 alp01 0 alp10 0 alp11 0; * parms statement to provide starting values;

parms Sig {.01 0 0 10} tau0 1 tau1 0

Sig0 {.01 0 0 10};

prior alp: ~ normal(0,var=1000); * prior distributions for fixed effects, weakly informative;

prior Sig ~ iwish(2,S); * prior distributions for covariance matrix, inverse Wishart;

prior Sig0 ~ iwish(2,S0); * prior distributions for covariance matrix, inverse Wishart;

prior tau0 tau1 ~ normal(0,var=1000); * prior distributions for effects that define the within-subject variance;

random pj ~ mvn(mu,Sig0) subject=pattern1; * pattern-level random intercept and slope with a covariance;

random theta ~ mvn(pj,Sig) subject=id; * subject-level random intercept and slope with a covariance;

f0 = alp00 + alp01*drug + b0i + k*pj0; * random intercept as a function of drug and k = 0 if no missing pattern and k = 1 if a missing pattern;

f1 = alp10 + alp11*drug + b1i + k*pj1; * random slope as a function of drug and k = 0 if no missing pattern and k = 1 if a missing pattern;

pred = f0 + f1*sqrt(wk); * predicted value of y;

s2e = exp(tau0 + tau1*drug); * exponential level-1 residual variance model as a function of drug;

LL = (-.5)*((((y-pred)**2)/s2e) + log(s2e) + log(2*pi)); * normal log-likelihood;

model y~ general(LL);

title1 "shared parameter model";

title2 "linear growth by sqrt week + heterogeneity of variance by drug";

proc mcmc data=all seed=465798789 nbi=200000 nmc=50000000 thin=500 outpost=outMixed plots=all;

array alp [4] alp00 alp01 alp10 alp11;

array theta[2] b0i b1i; * subject level random effects;

array mu[2] (0 0);

array pj[2] pj0 pj1; * pattern-level random effects;

array Sig[2,2];

array S[2,2] (.01 0 0 .01);

array Sig0[2,2];

array S0[2,2] (.01 0 0 .01);

beginnodata;

pi = arcos(-1);

endnodata;

parms alp00 0 alp01 0 alp10 0 alp11 0; * parms statement to provide starting values;

parms Sig {.01 0 0 10} tau0 1 tau1 0

Sig0 {.01 0 0 10};

prior alp: ~ normal(0,var=1000); * prior distributions for fixed effects, weakly informative;

prior Sig ~ iwish(2,S); * prior distributions for covariance matrix, inverse Wishart;

prior Sig0 ~ iwish(2,S0); * prior distributions for covariance matrix, inverse Wishart;

prior tau0 tau1 ~ normal(0,var=1000); * prior distributions for effects that define the within-subject variance;

random pj ~ mvn(mu,Sig0) subject=pattern1; * pattern-level random intercept and slope with a covariance;

random theta ~ mvn(pj,Sig) subject=id; * subject-level random intercept and slope with a covariance;

f0 = alp00 + alp01*drug + b0i + k*pj0; * random intercept as a function of drug and k = 0 if no missing pattern and k = 1 if a missing pattern;

f1 = alp10 + alp11*drug + b1i + k*pj1; * random slope as a function of drug and k = 0 if no missing pattern and k = 1 if a missing pattern;

if ind = 1 then do; * ind = 1 if y is mental illness rating;

pred = f0 + f1*sqrt(wk); * predicted value of y;

s2e = exp(tau0 + tau1*drug); * exponential level-1 residual variance model as a function of drug;

LL = (-.5)*((((y-pred)**2)/s2e) + log(s2e) + log(2*pi)); * normal log-likelihood;

end;

else if ind = 2 then do; * ind = 2 if y = ln(week last observed);

mu = gamma0 + gamma1*drug + gamma2*b0i + gamma3*b1i; * predict last week observed by drug, random intercept, random slope;

s2e2 = exp(ep0 + ep1*drug); * exponential level-1 residual variance model as a function of drug;

LL = (-.5)*((((y-mu)**2)/s2e2) + log(s2e2) + log(2*pi)); * normal log-likelihood;

end;

model y ~ general(LL);
