# Supplementary material for: Bayesian pattern-mixture models for dropout and intermittently missing data in longitudinal data analysis
Source: Behav Res Methods. 2023 May 23;56(3):1953–67. doi: 10.3758/s13428-023-02128-y (PMC10990982; doi:10.3758/s13428-023-02128-y)
Supplement: Supplementary file 3 — (DOCX 12 kb) [file 13428_2023_2128_MOESM3_ESM.docx]

Mplus script to simulate data

TITLE:

linear growth model

with missing data on a continuous outcome

y's are outcomes, x's covariates

x1 is a binary covariate that predicts

missingness and the random intercept and

slope; x1 is included in model fitted to

the data

x2 is a normally distributed covariate

that predicts missingness and the random

intercept and slope but is treated

as an 'unmeasured' covariate of the missingness

MONTECARLO:

NAMES ARE x1 x2 y1-y6;

NOBSERVATIONS = 400;

NREPS = 100;

SEED = 141;

GENERATE = x1(1);

CATEGORICAL = x1;

MISSING = y2-y6;

REPSAVE = ALL;

save = mnar_rep*.dat;

MODEL POPULATION:

[x1$1@0];

[x2@0]; x2@1;

i s | y1@0 y2@1 y3@2 y4@3 y5@4 y6@5;

[i*1 s*2];

i*1; s*.5;

i WITH s*.1;

y1-y6*.3;

i ON x1*.5 x2*1;

s ON x1*.2 x2*.5;

x1 on x2*.5;

MODEL MISSING:

[y2-y6@-1];

y2 ON x1*.2 x2*.3;

y3 ON x1*.4 x2*.6;

y4 ON x1*.8 x2*1.2;

y5 ON x1*1.6 x2*2.4;

y6 ON x1*3.2 x2*4.8;

ANALYSIS:

ESTIMATOR = ML;

MODEL:

i s | y1@0 y2@1 y3@2 y4@3 y5@4 y6@5;

[i*1 s*2];

i*1; s*.5;

i WITH s*.1;

y1-y6*.3;

i ON x1*.5 x2*1;

s ON x1*.2 x2*.5;

x1 on x2*.5;

x2@1;

[x1$1*0];

OUTPUT:

TECH9;
